# Efficacy of Connective Tissue Therapy and Abdominal Stretching Exercises in Individuals With Primary Dysmenorrhea: A Review

**DOI:** 10.7759/cureus.46553

**Published:** 2023-10-05

**Authors:** Dhanashree S Upganlawar, Shubhangi Patil, Prasad P Dhage

**Affiliations:** 1 Physiotherapy, Ravi Nair Physiotherapy College, Datta Meghe Institute of Medical Sciences, Wardha, IND; 2 Community Health Physiotherapy, Ravi Nair Physiotherapy College, Datta Meghe Institute of Medical Sciences, Wardha, IND

**Keywords:** quality of life, pain, connective tissue massage, abdominal stretching, primary dysmenorrhea

## Abstract

Dysmenorrhea is a menstrual disorder characterized by painful uterine cramps that occur during menstruation. There are two types of dysmenorrhea, primary and secondary. It affects 45-95% of all menstruating women worldwide. The prevalence in India is approximately 75%. Primary dysmenorrhea diagnosis is based on the patient's medical history and physical examination. If the history of start and duration of lower abdominal discomfort suggests secondary dysmenorrhoea or if the dysmenorrhoea does not respond to medical treatment, a pelvic examination is necessary to evaluate dysmenorrhoea. Because of the increasingly large number of women who are impacted by primary dysmenorrhea, it should be a public health concern that authorities must address. Abdominal stretching is a very simple, efficient, and risk-free workout. Some of the benefits of stretching exercises for dysmenorrhea include increased elasticity and strength of the spine and pelvic muscles and reduction in pain. The knee-to-chest exercise in combination with hydrocollator packs has a significant effect in improving the pain and the monthly irregularities in primary menstrual pain. Massage of connective tissue is a form of cutaneous stimulation that tries to stimulate the connective tissue's mechanical receptors. Connective tissue massage studies for treating a range of dysfunctions usually indicate that patients treated with this modality get pain alleviation and even complete remission.

## Introduction and background

Dysmenorrhea is a menstrual disorder characterised by painful uterine cramps that occur during menstruation. It's one of the most prevalent reasons for pelvic pain in adolescent and adult women, as well as school or job absences [1]. Dysmenorrhea is a frequent gynaecological condition that affects nearly half of all reproductive-age women. There are two classes of dysmenorrhea, primary and secondary. Primary dysmenorrhea (PD) is menstrual discomfort without pelvic disease that develops one to two years after menarche, at the same time as ovulation cycles stabilise, and lasts 48-72 hours every cycle [2]. Headache, backache, moodiness, irritability, and unpleasant urination and pain are all features of PD. Gastrointestinal (GI) symptoms such as nausea, bloating, diarrhoea, constipation, or both, as well as vomiting and indigestion, are all common symptoms of dysmenorrhea. Dysmenorrhea is also linked to fatigue and dizziness [3]. Secondary dysmenorrhea or menstrual discomfort originates from structural or very small pelvic disease, such as adenomyosis, or muscular tumour, or hereditary malformations of the pelvic reproductive organs, which are the most frequent causes [4].

It affects 45-95% of all menstruating women worldwide. The prevalence in India is approximately 75% [4]. The prevalence of dysmenorrhea was 84.2%, with roughly 34.2% of these girls reporting "severe pain," defined as a visual analogue score of 7 to 10 in 2015 [5]. According to a study published in 2018, the prevalence rate was 73.83% in 2018 among Indian universities [6]. In the article which was published, the prevalence rate was 60% among the Indian university [7]. The data show that every year, 140 million hours of work or school are lost due to stomach discomfort. Compared to their painless follicular stages and those painless and effortless during menstruation, women with PD report a significantly worse quality of life [4]. PD has a detrimental influence on intellectual and even psychosocial elements of livelihood for a substantial number of female adolescents, implying that failing to treat dysmenorrhea creates a significant social and financial burden for our close ones, neighbours, social groups, and the world at large [8]. Early menarche, null parity, irregular menstrual cycles, extended menstrual duration, high bleeding, heredity history of dysmenorrhea, and smoking have all been related to an increased risk of dysmenorrhea [9].

PD's pathophysiology is not fully known. Regardless, the uterine inner lining's hyper-secretion of prostaglandins has been established as the cause. Prostaglandin F2alpha (PGF-2a) and prostaglandin PGF 2 raise the uterine tone and generate uterine contractions with a large amplitude. In addition, PD has been linked to vasopressin. Vasopressin enhances uterine contractility and, due to its vasoconstriction effects, can produce ischemic discomfort [10]. Uterine contractility is more visible during the starting two days of the menstrual cycle. Before menstruation, progesterone levels drop, leading to an increase in prostaglandin (PG) production and dysmenorrhea. Endometriosis and adenomyosis are the most common factors which lead to secondary dysmenorrhea in premenopausal women [11]. Because of the increasingly large number of women who are impacted by PD, it should be considered a public health concern that authorities must address [12]. 

Although it is a prevalent ailment, it is frequently misdiagnosed, and most women do not seek medical help [13]. PD diagnosis is based on the patient's medical history and physical examination. If the history of start and duration of lower abdominal discomfort suggests secondary dysmenorrhoea or if the dysmenorrhoea does not respond to medical treatment, a pelvic examination is necessary to evaluate dysmenorrhoea [14]. The use of ultrasonography in the diagnosis of PD is of limited utility. Ultrasound, on the other hand, can help distinguish between secondary dysmenorrhea and reasons such as endometriosis and adenomyosis [15].

Material and methodology

This review article comprises valued research taken from 2008 to 2022 to explore the effect of abdominal stretching and connective tissue massage on relieving pain and the quality of life of women suffering from PD.

Data sources and search engine

For this review, data was extracted from the original articles, meta-analysis, review articles, and randomized control trials. For this review article Google Scholar, PubMed and Scopus were used to search the articles. Screening of the articles was also done by using keywords. For sorting out the articles following keywords were used: primary dysmenorrhea, abdominal stretching, connective tissue massage, pain, and quality of life.

## Review

Objective

Our objective was to present a summary of relevant literature on the effects of connective tissue massage and abdominal stretching exercises on pain and quality of life in individuals with PD.

Inclusion and exclusion criteria

**Figure 1:**
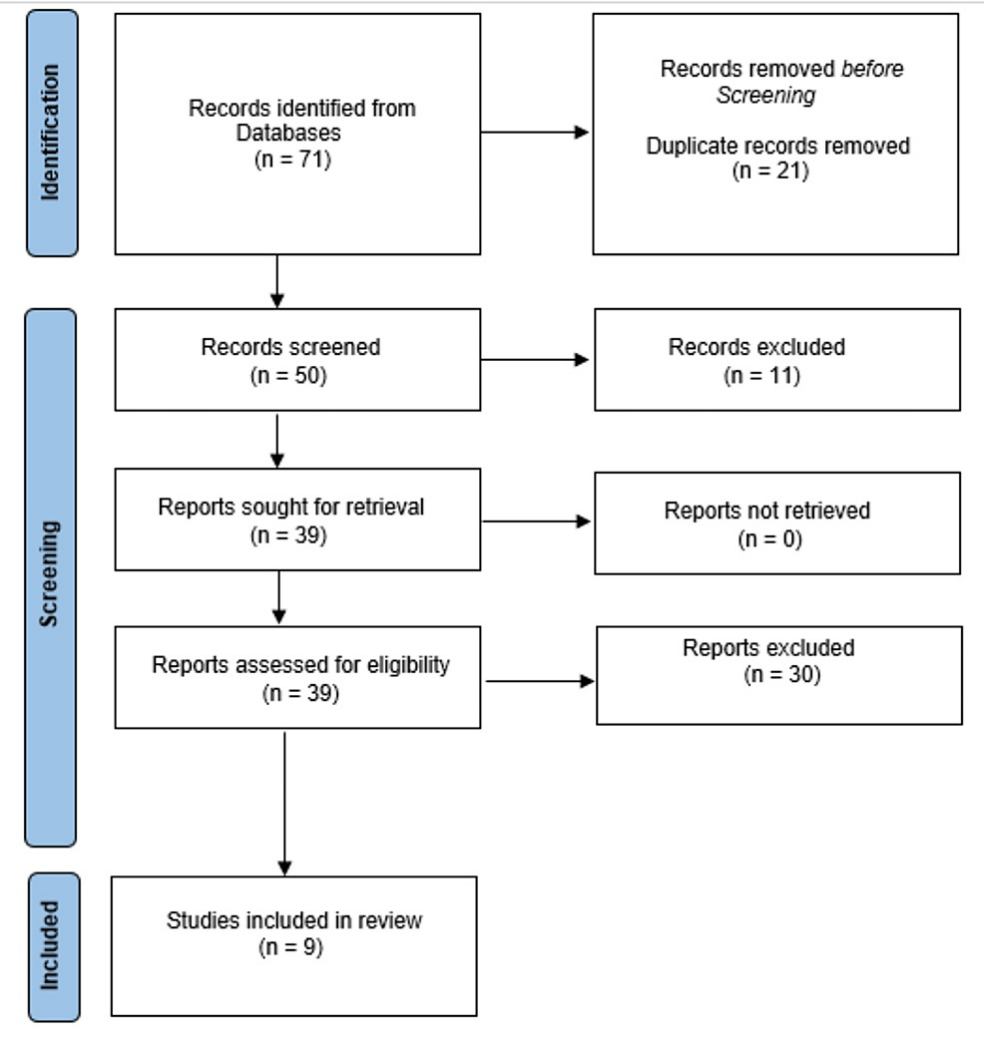
Preferred Reporting Items for Systematic Reviews and Meta-Analyses (PRISMA) Chart

The initial search yielded 136 relevant items. Following a thorough examination of the references, 54 were identified. After removing papers based on inclusion criteria, lack of full-text availability, or language difficulties, the study included 18 articles (Figure 1). One of the key reasons for the rejection of the articles was the inability to access the complete version of the articles.

Method 1: Abdominal Stretching 

Abdominal stretching is one type of non-pharmacological physical exercise that has a deliberate and intended physiological effect. Stretching the muscles, particularly the stomach muscles, for 10-15 minutes is referred to as abdominal stretching [16]. The findings show that exercise causes endorphins to be produced in the brain and spinal cord, which helps reduce pain [17]. Abdominal stretching is a very simple, efficient, and risk-free workout. Some of the advantages of stretching exercises for dysmenorrhea include increased adaptability and strength of the spine and pelvic floor muscles, the inspiratory muscle becoming more flexible and strong, oxygen supply and other fluids being transmitted properly to the uterus, decreased joint and back pain, stimulation of desire to eat and bowel action, anemia reduction and free passage during the menstrual cycle, and hormonal balance [18]. Forward bend through the hip joints, backward trunk bending, heel raise (bilateral), half squatting, trunk side flexion (bilateral), abdominal contraction, knee to chest (bilateral), hamstrings stretching (bilateral), pelvic bridging, plank, and calf stretching are some of the exercises [19].

Method 2: Connective Tissue Massage (CTM)

Connective tissue massage is a type of manual reflex therapy developed by Elizabeth Dicke in Germany in the late 1930s [20]. Dicke proposed the physiotherapeutic therapy of internal organs employing a massage technique that stimulates the corresponding cutaneous tissue with the goal of influencing the internal organ through reflex transmission. Massage of connective tissue is a form of cutaneous stimulation that tries to stimulate the connective tissue's mechanical receptors [21]. Massage of connective tissue is identified by applying a firm pressure to ligaments and subcutaneous tissues. Physical therapists utilise this approach to treat somatic or visceral illnesses [22]. Massage of connective tissue studies for the treatment of a range of dysfunctions usually indicate that patients who are treated with this modality get pain alleviation and even complete remission [23].

According to Head's theory, the sensory nerves transfer the stimulus to the spinal cord via the sympathetic ganglia. This stimulus would limit pain transmission by small-diameter fibres by releasing opiates like encephalin in the posterior root nerve of the spinal cord [24]. Massage of connective tissue is used on the basic section for dysfunctions involving the pelvic organs. The fundamental massage of connective tissue entails manipulating the following areas: sacrum, lumbar, and cervical, subcostal, lumbar, and final thoracic vertebrae [24].

Discussion

Physical activity such as stretching has been suggested as a non-pharmacological treatment option for certain symptoms that are caused by dysmenorrhea. Exercising appears to have analgesic properties that work in a nonspecific manner. Exercises also increase the synthesis of endorphins, which relieve the pain easily and consistently in the body. Period cramps can be relieved by applying the stretch to abdominal muscles [25]. For the management of PD in adolescents, abdominal stretching exercise is indicated as a safe measure. This activity is tremendously beneficial to young women and has now become a new regimen for them [26].

The primary goal of PD treatment is to alleviate pain and other associated symptoms (such as back and leg pain, anxiety, tension, and other symptoms that impair the quality of life) [27]. The researchers found that stretching the muscles of the abdomen and giving the relaxation technique to women who are suffering from menstrual cramps can relieve the symptoms when it is done regularly [28]. The results of an article published in 2017 state that after performing the exercise for a particular period of time the relieving of the symptoms of dysmenorrhea has increased with time [29].

According to research published in 2017, knee-to-chest exercise in combination with a hot moist pack (HMP) has a significant effect in improving the pain and the monthly irregularities in primary menstrual pain [30]. The abdominal muscle stretching exercise assists in improving the oxygenation process and also assists in lymphatic drainage by increasing the strength and flexibility of the abdominal muscle and help in reducing the menstrual cramps [31]. One study has found that there is a significant relationship between abdominal activity and reduction in the degrees of muscular fatigue, particularly in the abdomen, where pain intensity may be reduced [32]. Various studies have stated that regular physical activity is linked to a lower risk of dysmenorrhea [33].

In one study, one group received CTM and showed a remarkable decrease in pain and reduction in medicine use (Table 1) [34]. CTM strokes serve to move connective tissue by causing mechanical distortions. Connective tissue massage causes mast cells to produce histamine, and hence results in local swelling and arteriolar dilatation [35]. The dangerous substances are eliminated from the tissues, resulting in reduced inflammation and pain [36].

**Table 1:** Characteristics of key references.

Sr. no.	First author	Year published	Year conducted	Study location	Subject/sample	Type of study	Results
1	Murtiningsih [16]	2018	Not cleared	Indonesia	19 adolescent girls with dysmenorrhea	Interventional study	It proves that abdominal stretching exercises give great influences to reduce pain scale of dysmenorrhea in adolescent girls.
2	M. Nadjib Bustan [17]	2018		Indonesia	46 female nursing students	Quasi-experimental study	Abdominal stretching exercises suggested for young women to deal with primary dysmenorrhea in a non-drug manner.
3	Hend S Saleh [19]	2016	2012	Egypt	150 females with primary dysmenorrhea	Randomized control study	Active stretching exercises seems to be an easy, non-pharmacological method for managing primary dysmenorrhea.
4	Conceição Aparecida de Almeida Santos Reis [21]	2010		Brazil	72 women with primary dysmenorrhea	Pilot observational cohort study	Connective tissue massage may cause a reduction in menstrual pain.
5	Reda Mohamed-Nabil Aboushady [25]	2016		Saudi Arabia	80 dysmenorrhoeal students	A quasi–experimental design	Practicing the stretching exercises besides the usual menstrual care reduced the intensity of pain during menstruation
6	Sri Rezkiani Kas [26]	2020		Indonesia	30 females between 14-17 years of age	Quasi-experimental study	Abdominal stretching exercise three times before menstruation is more effective in reducing pain.
7	Narges Motahari-Tabari [29]	2016	2014	Iran	122 female students with moderate to severe dysmenorrhea	Randomized clinical trial	Regular exercise can be useful as an easy, accessible, and inexpensive approach to improve dysmenorrhea.
8	Ozgul [34]	2018	-	-	23 women with primary dysmenorrhea	Randomized controlled trial	Connective tissue massage (CTM) seems to be an effective approach in the short-term in primary dysmenorrhea (PD).

The CTM approach was able to significantly alleviate cramps in the lower abdomen area, as well as accompanying complaints such as low back pain. Every woman's progesterone level was found to be lower after physical therapy. Other researchers looked into the effectiveness of connective tissue manipulation as well and the result is shown to be effective in reducing pain [37]. Various physiotherapy techniques are useful in reducing menstrual pain and they also improve the quality of life of the individuals. Some of the physiotherapy techniques include yoga, connective tissue massage, abdominal stretching, Kinesio Taping, etc. [38]. 

Limitation and recommendation

Even if only two resources were used to scan the data for all published papers, it's possible that some important research would have gone unnoticed. Most publications were in English. Additionally, this research advocated the usage of this physiotherapy treatment to reduce pain and enhance people's quality of life.

## Conclusions

According to the results of many studies in this review, performing exercise in various forms including stretching exercises and connective tissue massage helps in reduction of pain intensity and duration of PD. Both techniques can be used safely as an alternative therapy for reduction of the pain.
